# Comparing ^18^F-AV-1451 with CSF t-tau and p-tau for diagnosis of Alzheimer disease

**DOI:** 10.1212/WNL.0000000000004887

**Published:** 2018-01-30

**Authors:** Niklas Mattsson, Ruben Smith, Olof Strandberg, Sebastian Palmqvist, Michael Schöll, Philip S. Insel, Douglas Hägerström, Tomas Ohlsson, Henrik Zetterberg, Kaj Blennow, Jonas Jögi, Oskar Hansson

**Affiliations:** From the Clinical Memory Research Unit (N.M., R.S., O.S., S.P., M.S., P.S.I., O.H.), Faculty of Medicine, Lund University; Memory Clinic (N.M., O.H.) and Departments of Neurology (N.M., R.S., S.P.), Clinical Neurophysiology (D.H.), Radiation Physics (T.O.), and Clinical Physiology and Nuclear Medicine (J.J.), Skåne University Hospital, Lund; MedTech West and the Department of Clinical Neuroscience (M.S.), University of Gothenburg, Sweden; Center for Imaging of Neurodegenerative Diseases (P.S.I.), Department of Veterans Affairs Medical Center, San Francisco; Department of Radiology and Biomedical Imaging (P.S.I.), University of California, San Francisco; Clinical Neurochemistry Laboratory (H.Z., K.B.), Sahlgrenska University Hospital; Institute of Neuroscience and Physiology, Department of Molecular Neuroscience (H.Z., K.B.), the Sahlgrenska Academy at the University of Gothenburg, Mölndal, Sweden; and Department of Molecular Neuroscience (H.Z.), UCL Institute of Neurology, Queen Square, London, UK.

## Abstract

**Objective:**

To compare PET imaging of tau pathology with CSF measurements (total tau [t-tau] and phosphorylated tau [p-tau]) in terms of diagnostic performance for Alzheimer disease (AD).

**Methods:**

We compared t-tau and p-tau and ^18^F-AV-1451 in 30 controls, 14 patients with prodromal AD, and 39 patients with Alzheimer dementia, recruited from the Swedish BioFINDER study. All patients with AD (prodromal and dementia) were screened for amyloid positivity using CSF β-amyloid 42. Retention of ^18^F-AV-1451 was measured in a priori specified regions, selected for known associations with tau pathology in AD.

**Results:**

Retention of ^18^F-AV-1451 was markedly elevated in Alzheimer dementia and moderately elevated in prodromal AD. CSF t-tau and p-tau was increased to similar levels in both AD dementia and prodromal AD. ^18^F-AV-1451 had very good diagnostic performance for Alzheimer dementia (area under the receiver operating characteristic curve [AUROC] ∼1.000), and was significantly better than t-tau (0.876), p-tau (0.890), hippocampal volume (0.824), and temporal cortical thickness (0.860). For prodromal AD, there were no significant AUROC differences between CSF tau and ^18^F-AV-1451 measures (0.836–0.939), but MRI measures had lower AUROCs (0.652–0.769).

**Conclusions:**

CSF tau and ^18^F-AV-1451 have equal performance in early clinical stages of AD, but ^18^F-AV-1451 is superior in the dementia stage, and exhibits close to perfect diagnostic performance for mild to moderate AD.

**Classification of evidence:**

This study provides Class III evidence that CSF tau and ^18^F-AV-1451 PET have similar performance in identifying early AD, and that ^18^F-AV-1451 PET is superior to CSF tau in identifying mild to moderate AD.

Alzheimer disease (AD) is characterized by the aggregation of β-amyloid (Aβ) in extracellular plaques and phosphorylated tau (p-tau) in intracellular neurofibrillary aggregates. Tau can be measured in CSF as total tau (t-tau), which is increased in AD and in several other neurologic diseases, or as p-tau, which is more specifically increased in AD.^1^ PET tracers have made it possible to visualize and quantify tau deposits in vivo. One of these tracers, ^18^F-AV-1451 (formerly called ^18^F-T807^2,3^), binds to tau aggregates in AD^4^ and differentiates AD from controls.^5–9^ Preliminary evidence indicates that CSF tau and PET tau measures correlate,^6,10^ but those results stem from populations mainly consisting of controls, including few cases with AD dementia. A key unresolved question is therefore if CSF and PET tau measures have similar or different diagnostic performance for AD. It is also not clear if CSF and PET tau measures are significantly better than MRI measures of brain structure to identify AD. To address these questions, we compared CSF t-tau and p-tau, ^18^F-AV-1451 PET, hippocampal volume, and cortical thickness in AD-associated regions^11^ for diagnosis of AD at the dementia and prodromal stages of the disease. All patients with prodromal AD and AD dementia were screened for amyloid positivity using CSF Aβ42.

## Methods

### Participants

The study population stemmed from 3 cohorts from the prospective and longitudinal Swedish BioFINDER study (biofinder.se). In the present study, we included 30 cognitively normal control participants. They were eligible for inclusion if they (1) were aged ≥60 years old, (2) scored 28–30 points on the Mini-Mental State Examination (MMSE) at the screening visit, (3) did not fulfill the criteria of mild cognitive impairment (MCI) or any dementia, and (4) were fluent in Swedish. The exclusion criteria were (1) presence of significant neurologic or psychiatric disease (e.g., stroke, Parkinson disease, multiple sclerosis, major depression), (2) significant systemic illness making it difficult to participate, (3) refusing lumbar puncture, or (4) substantial alcohol abuse. In the second cohort, 14 patients with MCI due to AD (prodromal AD) were enrolled at the Memory Clinic of the Skåne University Hospital, Sweden. These participants were eligible for inclusion if they (1) were referred to the memory clinics because of cognitive impairment, (2) did not fulfil the criteria for dementia, (3) scored 24–30 points on the MMSE, (4) had objective memory impairment according to delayed word list recall, (5) were aged 60–80 years, (6) had low CSF Aβ42 levels,^12^ and (7) were fluent in Swedish. The exclusion criteria were (1) cognitive impairment explained by another condition (other than prodromal dementia), (2) a substantial systemic illness making it difficult to participate, (3) refusing lumbar puncture, or (4) substantial alcohol abuse. In the last cohort, we included 39 patients with AD dementia at baseline, who were recruited at the Memory Clinic, Skåne University Hospital. All patients with dementia met the DSM-III-R criteria for dementia^13^ as well as the National Institute of Neurological and Communicative Disorders and Stroke–Alzheimer’s Disease and Related Disorders Association criteria for AD^14^ and had low CSF Aβ42 levels. The exclusion criteria were (1) substantial systemic illness making it difficult to participate or (2) substantial alcohol abuse. The diagnosis of prodromal AD and AD dementia were established by physicians specialized in dementia disorders, who were blinded to the ^18^F-AV-1451 PET, CSF t-tau, and CSF p-tau data.

### Cognitive measures

We used the MMSE as a measure of general cognition and the delayed recall memory test from the Alzheimer’s Disease Assessment Scale–cognitive subscale (list learning, 10 items) as a measure of memory.^15^

### CSF biomarkers

CSF samples were derived from lumbar puncture. Samples were analyzed at the Clinical Neurochemistry Laboratory in Mölndal, Sweden, for t-tau, p-tau, and Aβ42 using commercially available ELISAs (INNOTEST; Fujiribio, Ghent, Belgium). All CSF samples were analyzed using clinical practice procedures, with analyses performed by board-certified technicians blinded to clinical data, following detailed procedures to assure analytical precision and long-term stability of the biomarkers, including batch bridging between old and new batches of ELISA plates, general laboratory procedures (e.g., calibration of pipettes and preventive service of instruments), and strict criteria for approval of calibration curves and internal quality control (QC) samples, following the Westgard multi rules, as described previously in detail.^16^ The approval limits for the 2 internal QC CSF samples run at 2 positions on each plate was 12.0% for Aβ42, 9.3% for t-tau, and 9.8% for p-tau for the normal QC sample, and 11.0% for Aβ42, 10.0% for t-tau, and 9.8% for p-tau for the AD-like QC sample. For CSF Aβ42, we used a cutoff of <650 ng/L to identify Aβ-positive participants, based on our previous comparisons between CSF Aβ42 and Aβ PET imaging.^16^ All patients with prodromal AD and patients with AD dementia were screened for Aβ positivity before ^18^F-AV-1451 PET scanning. The control population was enriched for Aβ pathology, by inclusion of 15 Aβ-positive and 15 Aβ-negative participants before ^18^F-AV-1451 PET scanning.

### MRI and processing

T1-weighted imaging was performed on a 3T magnetic resonance scanner (Siemens Tim Trio 3T; Siemens Medical Solutions, Erlangen, Germany), producing a high-resolution anatomic magnetization-prepared rapid gradient echo image (repetition time 1,950 ms, echo time 3.4 ms, 1 mm isotropic voxels, and 178 slices) for further use in volumetric analysis, template normalization, and coregistrations. The anatomic scan was normalized to Montreal Neurological Institute 152 space^17^ with a diffeomorphic transform and the Advanced Normalization Tools (ANT) toolbox^18^ for further use in the PET processing pipeline (see below; ANT was used for all coregistrations). Cortical reconstruction and volumetric segmentation were performed with the Freesurfer image analysis pipeline v5.3 (surfer.nmr.mgh.harvard.edu/). We used the average cortical thickness in temporal lobe regions (including the FreeSurfer regions of interest [ROIs] entorhinal, fusiform, inferior temporal, and middle temporal cortex, based on reference 11), and hippocampal volume (averaged between right and left hemisphere).

### Tau PET imaging and processing

^18^F-AV-1451 was synthesized at Skåne University Hospital, Lund, as described previously.^19^ PET scans were performed on a GE Discovery 690 PET scanner (General Electric Medical Systems, Bensalem, PA) as dynamic scans using LIST-mode 80–120 minutes after a bolus injection of 370 MBq of ^18^F-AV-1451. Low-dose CT scans for attenuation correction were performed in the same patient position immediately prior to the PET scans. PET data were reconstructed into 5-minute frames using an iterative Vue Point HD algorithm with 6 subsets, 18 iterations with 3-mm filter, and no time-of-flight correction. The dynamic scans were motion corrected using AFNI's 3dvolreg,^20^ time-averaged and rigidly coregistered to the skull-stripped MRI scan. Partial volume error correction was performed using the geometric transfer method as described in reference 21 using the FreeSurfer parcellations, smoothed with 5-mm full width at half maximum to calculate transfers across ROI borders. The FreeSurfer parcellation in the magnetic resonance space of the anatomic scan was then applied to the processed, coregistered, and time-averaged PET image to extract regional uptake values. We created ^18^F-AV-1451 standardized uptake value (SUV) images based on mean uptake over 80–120 minutes postinjection normalized to uptake in a gray matter–masked cerebellum reference region to create voxelwise SUV ratio (SUVR) images in each participant's MRI native space.^19^

### Regional PET analyses

We performed ^18^F-AV-1451 PET analyses with a priori defined ROI, as proposed by Cho et al.^22^ and Jack et al.^23^ Cho et al.^22^ described a protocol to aggregate FreeSurfer ROIs in different tau stages, which was overall similar to the staging system suggested by Braak and Braak.^24^ We used this protocol to define a set of nonoverlapping ROIs corresponding to tau stages I–II, III, IV, V, and VI (table 1). To obtain an overall ^18^F-AV-1451 PET tau measure, we merged the signal from tau stages I–V. As an alternative overall tau measure, we merged the signal from tau stages regions I–IV, which corresponds to an aggregation protocol suggested by Jack et al.^23^ For merged regions, the signal was calculated as the sum of the volume-adjusted regional ^18^F-AV-1451 PET signals.

**Table 1:**
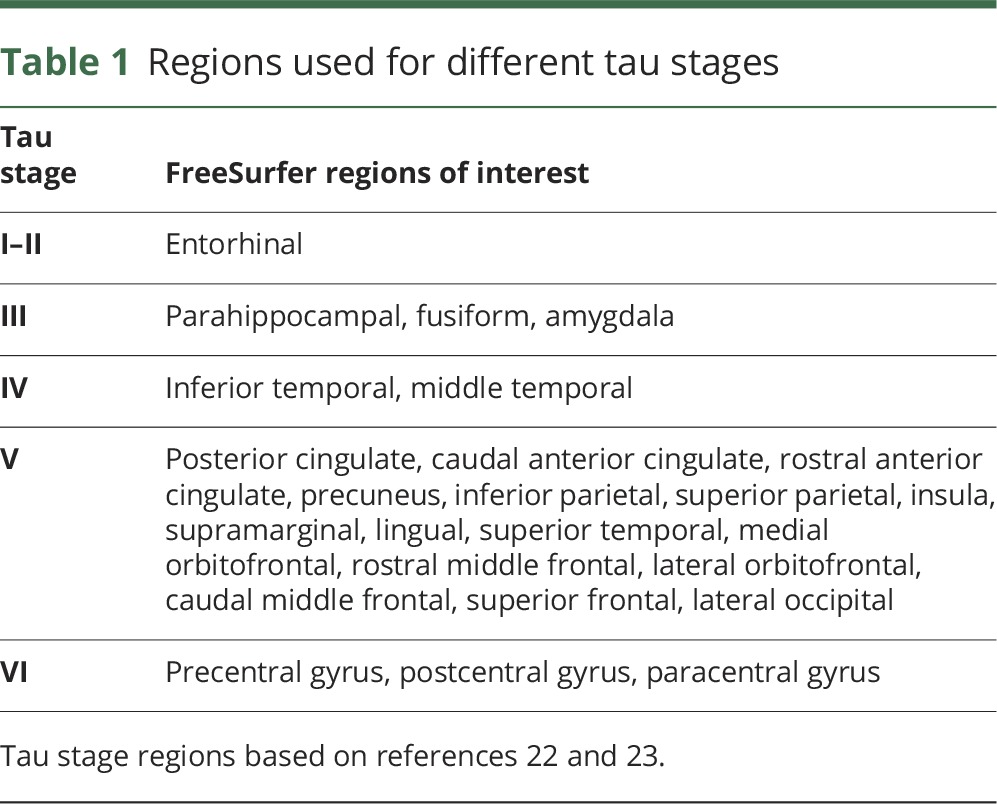
Regions used for different tau stages

### Statistical analyses

Biomarker levels were compared between diagnostic groups by linear regression, adjusted for age. Sex was also explored as a covariate, but was left out since it was nonsignificant and did not affect the results.

Diagnostic performance of biomarkers was quantified by area under the ROC (AUROC) analysis. AUROCs were compared between biomarkers using a bootstrap method with 2,000 iterations. For all tau biomarkers, we determined cutoffs by the Youden index (J, which maximizes the combination of sensitivity and specificity). We calculated sensitivities, specificities, and overall accuracies (proportion of correctly classified participants) at these cutoffs.

All statistical tests were 2-sided. Significance was determined at *p* < 0.05. All statistics were done using R (v. 3.2.3, The R Foundation for Statistical Computing). The pROC package (v.1.8) was used for AUROC analyses.

### Primary research questions

Do the diagnostic performances differ between CSF tau measures and ^18^F-AV-1451 PET for early AD (prodromal disease stage) and for AD dementia (mild to moderate disease stage)? This study provides Class III evidence that CSF tau measures and ^18^F-AV-1451 PET have similar performance for identifying early (prodromal) AD, and that ^18^F-AV-1451 PET is superior to CSF tau measures in identifying mild to moderate AD.

### Standard protocol approvals, registrations, and patient consents

All participants gave written informed consent to participate in the study. Ethical approval was given by the Ethical Committee of Lund University, Sweden, and all the methods were carried out in accordance with the approved guidelines. ^18^F-AV-1451 PET imaging approval was obtained from the Swedish Medicines and Products Agency and the local Radiation Safety Committee at Skåne University Hospital, Sweden.

## Results

### ^18^F-AV-1451, CSF tau, and MRI biomarkers by diagnosis

**Figure 1:**
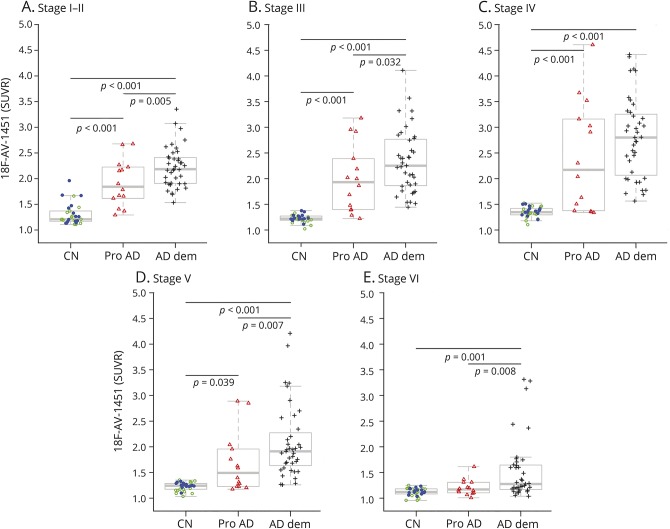
^18^F-AV-1451 by clinical diagnosis (A–E) ^18^F-AV-1451 signal in different tau stage regions. Diagnostic groups (controls [CN], prodromal Alzheimer disease [Pro AD], and Alzheimer disease dementia [AD dem]) were compared by linear regression, adjusted for age. The controls are coded by amyloid status (amyloid-negative, green open circles; amyloid-positive, blue dots). SUVR = standardized uptake value ratio.

**Figure 2:**
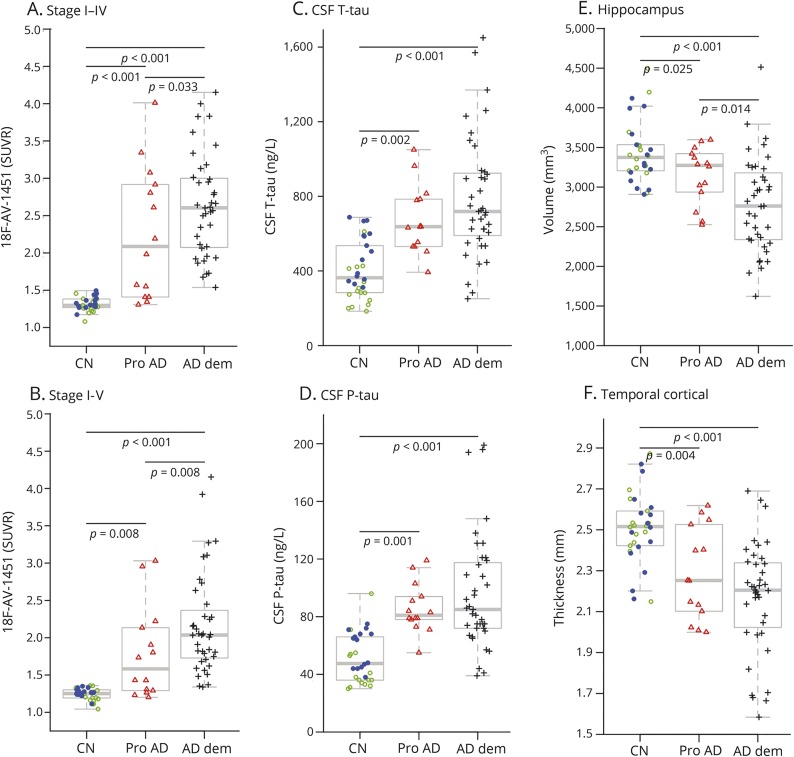
^18^F-AV-1451, CSF tau biomarkers, and brain structure (A, B) ^18^F-AV-1451 signal in tau stage regions I–IV and tau stage I–V. (C, D) CSF total tau (t-tau) and phosphorylated tau (p-tau). (E, F) Hippocampal volume and cortical thickness in temporal lobe regions. Diagnostic groups (controls [CN], prodromal Alzheimer disease [Pro AD], and Alzheimer disease dementia [AD dem]) were compared by linear regression, adjusted for age. The controls are coded by amyloid status (amyloid-negative, green open circles; amyloid-positive, blue dots).

Demographics are presented in table 2. Compared to controls, the ^18^F-AV-1451 retention was elevated in AD dementia in all tau stages, and in prodromal AD in tau stage I–V regions (figure 1). The ^18^F-AV-1451 retention was also elevated in AD dementia compared to prodromal AD in all tau stages except stage IV. The differences between the diagnostic groups in ^18^F-AV-1451 were similar for the merged stage I–IV and I–V regions (figure 2, A and B). Patients with AD dementia and prodromal AD had higher CSF t-tau and p-tau than controls, but there were no differences between patients with AD dementia and patients with prodromal AD in CSF tau measures (figure 2, C and D). Patients with AD dementia and prodromal AD had smaller hippocampi and thinner cortical thickness of the temporal lobe than controls, and patients with AD dementia had smaller hippocampi than patients with prodromal AD (figure 2, E and F).

**Table 2:**
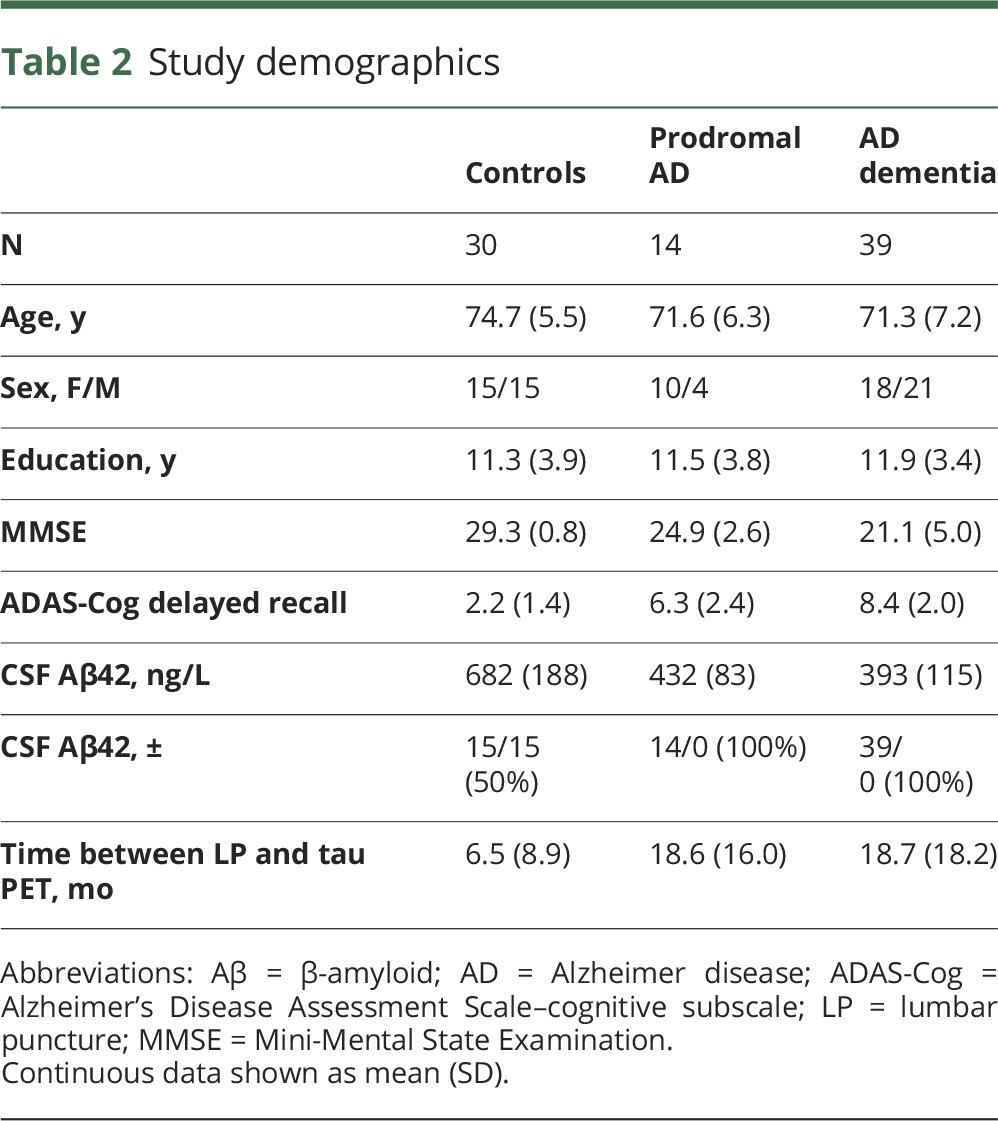
Study demographics

### Diagnostic performances of tau biomarkers

**Figure 3:**
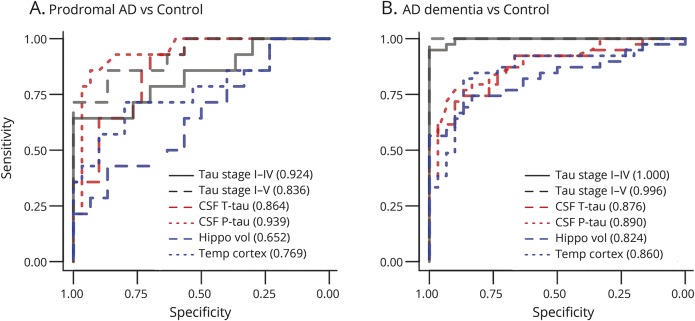
Area under the receiver operating characteristic curve (AUROC) analyses AUROC analyses for the ^18^F-AV-1451 signal from the tau stage region I–IV, tau stage region I–V, CSF total tau (t-tau) and phosphorylated tau (p-tau), hippocampal volume, and temporal lobe cortical thickness, to differentiate prodromal Alzheimer disease (AD) (A) and AD dementia (B) from controls. AUROCs are shown in the legends. AUROCs for hippocampal volume were adjusted for intracranial volume. For prodromal AD vs controls, tau stage I–IV, CSF t-tau, and CSF p-tau had higher AUROCs than hippocampal volume (*p* = 0.0055; *p* = 0.024; *p* = 0.0065). For AD dementia vs controls, tau stage I–V and tau stage I–IV PET had higher AUROCs than CSF t-tau (*p* = 0.0033; *p* = 0.0025), CSF p-tau (*p* = 0.0040; *p* = 0.0038), hippocampal volume (*p* < 0.001; *p* < 0.001), and temporal lobe cortical thickness (*p* = 0.0034; *p* = 0.0038).

We calculated AUROCs for ^18^F-AV-1451 in tau stage I–IV and stage I–V, CSF t-tau and p-tau, hippocampal volume, and temporal cortical thickness (figure 3). ^18^F-AV-1451 had almost perfect separation for AD dementia vs controls. The AUROCs were significantly higher for ^18^F-AV-1451 measures than for CSF t-tau, p-tau, and MRI measures, but there were no significant differences in AUROCs between CSF T-tau, p-tau, and MRI measures. For patients with prodromal AD vs controls, there were no significant differences in AUROCs between the tau biomarkers, but ^18^F-AV-1451 in tau stage I–IV, CSF t-tau, and CSF p-tau all had significantly higher AUROCs than hippocampal volume. The PET and CSF tau biomarkers also tended to have higher AUROCs than temporal lobe cortical thickness for prodromal AD, but the differences were not significant. The AUROC results were similar when not performing partial volume correction of the ^18^F-AV-1451 images, and when adjusting for age and time lag between PET and lumbar puncture (data not shown). We also compared biomarker AUROCS for preclinical AD (Aβ-positive controls, n = 15) vs prodromal AD and AD dementia. The findings were similar to when including all controls (supplementary analysis [links.lww.com/WNL/A93] and figure e-1, [links.lww.com/WNL/A91]).

We next determined optimal cutoffs for the PET and CSF tau biomarkers using the Youden index. For patients with AD dementia vs controls, these cutoffs were CSF t-tau >624 ng/L (sensitivity 69%, specificity 90%), CSF p-tau >72 ng/L (sensitivity 72%, specificity 93%), ^18^F-AV-1451 tau stage I–IV >1.54 SUVR (sensitivity 97%, specificity 100%), and ^18^F-AV-1451 tau stage I–V >1.37 SUVR (sensitivity 92%, specificity 100%). For prodromal AD vs controls, the cutoffs were CSF t-tau >504 ng/L (sensitivity 86%, specificity 70%), CSF p-tau >73 ng/L (sensitivity 79%, specificity 93%), ^18^F-AV-1451 tau stage I–IV >1.41 SUVR (sensitivity 79%, specificity 87%), and ^18^F-AV-1451 tau stage I–V >1.43 SUVR (sensitivity 57%, specificity 100%). See table e-1 (links.lww.com/WNL/A92) for a summary of these data.

## Discussion

We found that ^18^F-AV-1451 tau PET imaging was superior to CSF tau biomarkers for diagnosis of mild to moderate AD dementia vs controls, with almost perfect separation between groups. In prodromal AD, when some patients still lacked widespread tau pathology, ^18^F-AV-1451 PET and CSF tau biomarkers had comparable diagnostic performance.

Studies comparing CSF tau biomarkers with PET tau imaging for diagnosis of AD are rare. Our findings suggest that the relationship between CSF and PET tau biomarkers for diagnosis differs by disease stage in AD. This supports a model where CSF tau biomarkers are primarily useful as disease state biomarker, i.e., they indicate presence or absence of AD, but they may be less useful as stage biomarkers during the transition from prodromal AD to dementia. In contrast, ^18^F-AV-1451 imaging may be useful both as a state and a stage biomarker, since increased ^18^F-AV-1451 is associated with AD already at the prodromal stage, and provides increased separation towards controls in the dementia stage of the disease. We also included MRI measures of brain structure (hippocampal volume and temporal lobe cortical thickness), which had lower AUROC than ^18^F-AV-1451 for AD dementia. For prodromal AD, hippocampal volume had significantly lower AUROC than PET and CSF tau measures, and there was also a tendency for lower AUROC for temporal lobe cortical thickness compared to the tau measures.

At the dementia stage, ^18^F-AV-1451 was superior to CSF tau biomarkers for AD diagnosis. The diagnostic performance of CSF tau biomarkers may be confounded both by the physiologic between-person variability in CSF tau concentrations and by release of tau due to nonspecific neuronal injury.^25^ Another possibility that needs to be tested by longitudinal studies is that CSF tau may be more sensitive than ^18^F-AV-1451 to very early pathologic tau-related changes. For example, release of neuronal tau may be involved in interneuronal transmission of tau pathology,^26^ which hypothetically may occur before tau pathology is detected by ^18^F-AV-1451 imaging. Similarly, we have previously shown that CSF biomarkers may be more sensitive to Aβ pathology compared to PET imaging.^27^ The fact that CSF tau measures did not differ between prodromal AD and AD dementia suggests that these biomarkers plateau at the prodromal stage of the disease. In contrast, the ^18^F-AV-1451 signal was higher in the AD dementia than in the prodromal AD group, which likely reflects a continuous accumulation of tau as the disease progresses. One important difference between CSF and PET tau measurements is that ^18^F-AV-1451 makes it possible to track a potential spread of tau to new brain regions. Some regions may be affected later in the disease process (e.g., tau stage VI regions may be affected after tau stage V regions). This may explain why the latest stages show less separation between diagnostic groups than the earlier stages.

We did not find different results for CSF t-tau and p-tau, despite the fact that CSF p-tau has been suggested to be more closely related to brain tau pathology than CSF t-tau.^1^ However, we note that histopathology studies have found correlations for both CSF t-tau and p-tau with tangle load,^28–30^ which is in agreement with our finding that both CSF t-tau and p-tau had similar diagnostic performance as ^18^F-AV-1451.

One limitation is the lack of neuropathologic confirmation of tau pathology. Previous studies have found strong correlations between ^18^F-AV-1451 PET and tau aggregates consisting of combined 4R and 3R tau,^31^ and some studies have found correlations between CSF tau and brain tau pathology^28–30^ (but not all studies have confirmed this^32^). Another limitation is that we only included patients with prodromal AD and patients with AD dementia with biomarker evidence of amyloid pathology. This was done because modern research criteria emphasize amyloid biomarkers for a diagnosis of AD,^33,34^ but we acknowledge that it restricts the generalizability to patients with AD with evidence of amyloid pathology. Future studies may include a more diverse patient population, including patients with other dementias. We also acknowledge that both CSF tau and ^18^F-AV-1451 may be susceptible to measurement errors. The CSF tau measurements were done according to the Alzheimer's Association's guidelines, and the coefficients of variation were <10%. The ^18^F-AV-1451 signal may be susceptible to off-target binding in several locations. One prominent off-target site is the choroid plexus, which is very close to the hippocampal formation. Partly because of this, we did not include the ^18^F-AV-1451 signal in the hippocampus in this study.

We used the cerebellar gray matter as reference region for ^18^F-AV-1451, but the choice of an optimal reference region is not straightforward. There may be minor off-target binding of ^18^F-AV-1451 in the rostral part of cerebellum, which may decrease the ability of ^18^F-AV-1451 to separate patients with AD from controls. However, despite this potential source of variability, we demonstrated almost 100% separation between AD dementia and controls. In our view, this provides proof-of-principle of the superior diagnostic performance of ^18^F-AV-1451 compared to CSF tau measures. Other options for reference regions are problematic. Cerebellar white matter may have a relatively strong nonspecific binding. Cerebral white matter could be used, but the included ROIs then need to be clearly separated from the off-target binding regions in the basal ganglia/thalamus. Future studies may evaluate whether other reference regions would improve the results further.

We found that ^18^F-AV-1451 PET imaging has superior diagnostic performance compared to CSF tau for AD in the dementia stage, but the 2 tau biomarker modalities have equal performance for prodromal AD. Future studies may compare longitudinal CSF and PET tau measures to clarify how these measures may develop over time, and how they may respond to disease-modifying treatment in AD.

## Glossary

Aβ: β-amyloid
AD: Alzheimer disease
ANT: Advanced Normalization Tools
AUROC: area under the receiver operating characteristic curve
DSM-III-R: *Diagnostic and Statistical Manual of Mental Disorders, 3rd edition, revised*
MCI: mild cognitive impairment
MMSE: Mini-Mental State Examination
p-tau: phosphorylated tau
QC: quality control
ROI: region of interest
SUV: standardized uptake value
SUVR: standardized uptake value ratio
t-tau: total tau
